# Algorithm for pediatric orbital blowout fractures: a 20-year retrospective cohort study

**DOI:** 10.1016/j.bjorl.2023.01.004

**Published:** 2023-01-27

**Authors:** Kosuke Takabayashi, Yohei Maeda, Nobuya Kataoka, Hiroyuki Kagokawad, Masayoshi Nagaminea, Isao Otad, Taketoshi Fujita

**Affiliations:** a Japanese Red Cross Asahikawa Hospital, Department of Otorhinolaryngology, Asahikawa City, Hokkaido, Japan; b Osaka University Graduate School of Medicine, Department of Otorhinolaryngology Head and Neck Surgery, Suita City, Osaka, Japan; c Japan Community Health Care Organization Osaka Hospital, Department of Otorhinolaryngology, Osaka City, Osaka, Japan; d Japanese Red Cross Asahikawa Hospital, Department of Ophthalmology, Asahikawa City, Hokkaido, Japan

**Keywords:** Linear type fracture, Missing rectus, Oculocardiac reflex, Hess area ratio, Diplopia

## Abstract

**Objective:**

Pediatric orbital blowout fractures can include pathologies that seem mild but require urgent release; serious sequelae can occur with standby surgery or conservative treatment. We sought to validate an algorithm for the treatment of pediatric orbital blowout fractures.

**Methods:**

This retrospective cohort study included 61 pediatric patients, aged 18 years or younger, treated for pure orbital blowout fractures according to the algorithm from April 1, 2000, to August 31, 2020, at the Japanese Red Cross Asahikawa Hospital.

**Results:**

There were 52 males (85%). Median age was 14 years (range, 5–18 years). There were 9 patients categorized as needing urgent release, 16 as needing repair, and 36 as needing conservative treatment. Mean follow-up ocular movement was 98.0 (95% Confidence Interval [95% CI], 96.8–99.2). Postoperative diplopia was not observed in 96% (79.6%–99.9%) of patients, better than in previous studies. A higher proportion of patients aged 0–12 years needed urgent repair than those aged 13–18 years (Odds Ratio [OR] = 14.2; 95% CI 1.6–683.4; *p* = 0.0046). There were no differences in Hess area ratio by age group.

**Conclusion:**

Clinical results with the algorithm were satisfactory. The algorithm is suitable for treatment of pediatric orbital blowout fractures.

**Level of evidence:**

4.

## Highlights

• Develop a treatment algorithm for pediatric orbital blowout fractures.
• Findings to consider urgent release for linear type of pediatric orbital blowout fractures: missing rectus and OCR.
• Present the results using the Hess area ratio, an objective measure of treatment outcome.
• Simple algorithm easily used even for physicians who do not specialize in orbital care.
• Algorithm producing successful treatment outcomes.

## Introduction

Pediatric orbital blowout fractures are often associated with residual diplopia if not treated emergently. Pediatric bones are more flexible than adult bones; thus, pediatric blowout fractures occasionally result in linear fractures entrapping orbital contents, called trapdoor fractures,^1^, ^2^, ^3^, ^4^, ^5^ which occasionally need urgent surgical reduction. Over the past decade, surgical results, which have varied across reports, include persistent diplopia in 0%–55% of patients with pediatric blowout fractures.^1^, ^3^, ^6^, ^7^, ^8^, ^9^, ^10^, ^11^, ^12^, ^13^, ^14^, ^15^, ^16^, ^17^, ^18^, ^19^, ^20^, ^21^, ^22^, ^23^, ^24^, ^25^, ^26^, ^27^

The timing of surgery for urgent cases and the types of pathology requiring urgent surgery have been controversial. Previous studies have recommended surgery as soon as possible for urgent cases,^9^, ^10^, ^16^, ^20^ within 24 h,^1^, ^7^, ^13^ within 48 h,^2^, ^4^, ^28^ or over 48 h from injury.^11^, ^12^, ^17^, ^18^^,^^23^, ^29^ Previous studies have not described any definite algorithm for surgical intervention.

Regarding patient age, it has been reported that younger pediatric patients are less likely to have improvements in ocular movement than older pediatric patients.^30^ Younger pediatric patients were reported to have a higher rate of urgent cases.^6^

In previous studies, ocular movement was evaluated based on the presence of diplopia^3^, ^7^, ^8^, ^10^^,^^12^, ^14^, ^15^, ^16^, ^17^, ^19^, ^20^, ^21^, ^22^, ^24^, ^27^ or quantitative scales.^9^, ^11^, ^13^, ^18^^,^^26^ On the other hand, Hess Area Ratio (HAR%)^31^ is a definitive objective scale, but no previous studies of the clinical results of pediatric orbital blowout fractures have used an objective scale.

Here we present an algorithm for surgical intervention to treat pediatric orbital blowout fractures. This study is the first in which ocular movement was described based on both HAR% and the presence of diplopia. Ocular movement results after treatment according to the algorithm were subjectively and objectively satisfactory. The purpose of this study is to retrospectively examine the validity of our proposed algorithm by analyzing our clinical results according to the algorithm and comparing the results with historical controls in previous reports.^3^, ^6^, ^8^, ^11^^,^^12^, ^14^, ^20^, ^27^^,^^28^, ^32^

## Methods

### Study design

This study is a single-center retrospective cohort study designed to investigate the validity of the algorithm for the treatment of pediatric orbital blowout fractures.

### Algorithm and study population

**Figure 1:**
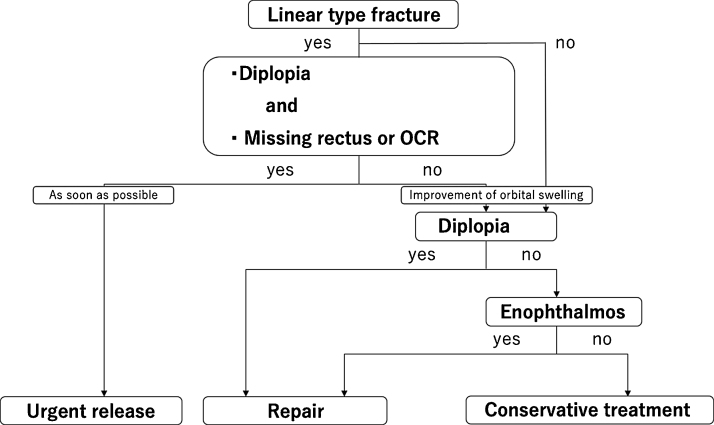
Algorithm for the treatment of orbital blowout fractures in pediatric patients. Patients are divided into 3 groups: urgent release, repair, and conservative treatment. OCR, Oculocardiac Reflex.

The study population consisted of patients aged less than 18 years who had orbital blowout fractures treated between April 2000 and August 2020 by our department. They were divided into the surgery group and the conservative treatment group. All patients were treated according to the algorithm (Fig. 1). Inclusion criteria included pure orbital blowout fracture for which we were able to follow the patient until ocular movement became stable and was evaluated objectively or subjectively. Exclusion criteria included ocular injury, optic nerve disorder, concomitant facial fractures, lack of follow-up until the symptomatology became stable, and lack of applicable data such as fracture area, fracture type, or HAR%.

In all patients, fracture area and type were initially assessed with Computed Tomography (CT). Patients were referred to an ophthalmologist for a comprehensive evaluation of the eyes. An otorhinolaryngologist and an ophthalmologist jointly assessed for missing rectus^9^, ^33^ or Oculocardiac Reflex (OCR).^1^, ^34^, ^35^, ^36^ Subsequently, the patient’s categorization was determined according to the algorithm.

#### Urgent release

Linear fractures associated with diplopia and missing rectus, or OCR were classified as urgent release, even if a HESS screen test was not performed. Patients underwent urgent surgical release as soon as possible.

#### Repair

Non-linear fractures and linear fractures not associated with diplopia, missing rectus, or OCR were classified as non-urgent. Fractures associated with diplopia or enophthalmos after complete resolution of orbital swelling were classified as repair. Such patients underwent non-urgent surgical repair. Awareness of diplopia within 30 degrees on the Hess screen test and subjective enophthalmos were considered indications for surgical repair.

#### Conservative treatment

Other types of cases or parental refusal of surgical treatment did not undergo surgical treatment. They were classified as conservative treatment. They received conservative treatments such as pain medications, oral antibiotics, and ocular movement rehabilitation.

After treatment, patients were followed by an otorhinolaryngologist and an ophthalmologist until the improvement in ocular movement became stable.

### Surgical techniques

All surgical treatments were performed under general anesthesia. The transorbital approach through a subciliary incision was used for linear fractures in the inferior wall. The transnasal endoscopic approach was used for linear fractures in the medial wall or non-linear fractures. All incarcerated orbital contents were placed back into the orbit. Reconstructive plates were not needed for linear fractures in the inferior wall. A balloon in the maxillary sinus or a silastic sheet in the ethmoid sinus was used for fixation as needed for all fracture types except for linear fractures in the inferior wall.

### Outcomes

The interval from injury to the start of surgery in the urgent release and repair groups was investigated. Ocular movements of patients who underwent surgery versus conservative treatment were compared. In addition, participants were divided into two groups: age 0–12 years and age 13–18 years. The proportion of urgent release and ocular movement of the two groups were compared. Finally, the proportion of patients without diplopia when ocular movement status became stable was compared to the proportion in all previous case series of pediatric orbital blowout fractures published after 2001, which were identified via a PubMed search with the terms “pediatric”, “blowout”, and “fracture” performed on May 31, 2021.

### Adverse events

Decreases in visual acuity, postoperative rhinosinusitis, and secondary injuries associated with surgical treatment were evaluated as adverse events.

### Measurements

We assessed all patients for ocular movement objectively using HAR%.^31^ HAR% is defined as the ratio of the area at the 30 degrees line on the affected side to the area on the unaffected side on the HESS chart. HAR% higher than 85 was defined as a good outcome, corresponding to no perception of diplopia. Ocular movement was assessed subjectively based on the presence or absence of diplopia, which was defined as the patient’s awareness of diplopia in daily life.

### Data source

Clinical data in this study were extracted during chart review.

### Bias

Selection bias and information bias in previous studies cannot be ruled out.

### Statistical analysis

Fisher’s exact test was used to compare the proportion of urgent cases among patients in the age 0–12 years and age 13–18 years groups. The Mann–Whitney *U* test was used to compare follow-up HAR% scores. Results are expressed as means (SD). Values of *p* < 0.05 were considered significant. All statistical analyses were performed with EZR,^37^ which is a modified version of the R commander designed to add statistical functions frequently used in biostatistics.

## Results

### Patients

**Figure 2:**
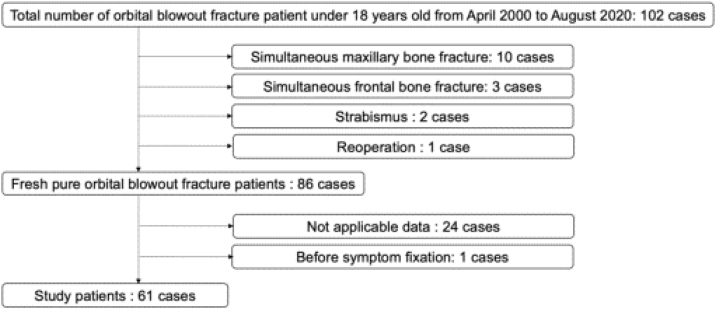
Patient flow diagram. Of 102 patients, 16 were excluded because of concomitant conditions. Of 86 patients with pure orbital blowout fractures, 25 were excluded because of insufficient data or symptoms that have not yet stabilized.

As shown in the study flow diagram (Fig. 2), 38 of 108 patients underwent surgery and 64 of 108 received conservative treatment. We excluded 10 patients with concurrent maxillary bone fractures, 3 patients with concurrent frontal bone fractures, 2 patients with strabismus diagnosed before injury, and 1 patient with reoperation. Next, we excluded 24 patients without applicable data and 1 patient whose symptoms had not yet stabilized. Finally, 61 patients were included in this study: 25 were in the surgery group and 36 were in the conservative treatment group. There were no cases of conservative treatment due to refusal of surgery. All 61 patients were properly categorized according to the algorithm.

### Patient categorization according to the algorithm

Patient categorization according to the algorithm was described in the Supplementary Materials.

### Interval from injury to the start of surgery

Among patients who underwent urgent release, the mean interval from injury to the start of surgery was 11.78 ± 7.74 h (range, 4–24 h). The mean interval among patients categorized as repair was 11.81 ± 5.60 days (range, 5–26 days).

## Patient demographics

Table 1 shows the characteristics of the study patients.

**Table 1:** Characteristics of the study participants.

Characteristic	Overall		Surgery		Conservative treatment	
	Value	(%)	Value	(%)	Value	(%)
Sex						
Male	52	(85)	20	(80)	32	(89)
Female	9	(15)	5	(20)	4	(11)
Age, years						
Median	14		13		15	
Range	5‒18		7‒18		5‒18	
Follow-up duration, days						
Mean	122.8		242.9		39.4	
Range	1‒922		27‒922		1‒587	
Decreased initial visual acuity	0/61	(0)				
Enophthalmos on presentation	1/61	(2)				
Enophthalmos during follow-up	0/61	(0)				
Surgical management	25/61	(41)				
Missing rectus			7/25	(28)	0/36	(0)
OCR			8/25	(32)	1/36	(3)
Urgent release			9/25	(36)		
Diplopia during follow-up			1/25	(4)	0/36	(0)
HAR% during follow-up						
Mean	98.0		96.0		99.4	
95% CI	96.8‒98.8		93.2‒98.8		98.8‒100.0	

OCR, Oculocardiac Reflex; HAR%, Hess Area Ratio; 95% CI, Confidence Interval.

### Overall

Of the 61 patients, 52 were male and 9 were female. Median age was 14 years, with a range of 5–18 years. Mean follow-up duration was 122.8 days (range, 1–922 days). None of the patients had symptoms such as decreased visual acuity initially or enophthalmos on follow-up, except for 1 patient who presented with enophthalmos. Mean HAR% on follow-up was 98.0 (95% Confidence Interval [95% CI] 96.8–99.2).

### Patients who underwent surgery

Overall, 25 patients underwent surgery. There were 7 patients with missing rectus and 8 with OCR, of whom 5 patients had both missing rectus and OCR, which are urgent signs in our algorithm. Two patients had only missing rectus and 3 patients had only OCR. None had postoperative diplopia.

### Patients who received conservative treatment

In total, 36 patients were followed without surgery. None had missing rectus; only 1 patient had OCR. None had diplopia during follow-up.

### Age 0–12 years group

eTable 1 in the supplement shows the characteristics of patients in the age 0–12 years group. The characteristics of the age 0–12 years group were described in the supplementary materials.

### Age 13–18 years group

eTable 2 in the supplement shows the characteristics of patients in the age 13–18 years group. The characteristics of the age 13–18 years group were described in the supplementary materials.

### Comparison of ocular movement during follow-up between patients who underwent surgery versus conservative treatment (Table 2)

There was a statistically significant difference in follow-up HAR% among patients who underwent surgery (mean = 96.0; 95% CI 93.2–98.8) versus conservative treatment (99.4; 98.8–100.0) (*p* = 0.0231). There were no statistically significant differences in follow-up HAR% in the age 0–12 years group among patients who underwent surgery (97.6; 84.8–100.4) versus conservative treatment (99.7; 99.5–99.9) (*p* = 0.125). There were no statistically significant differences in follow-up HAR% in the age 13–18 years group among patients who underwent surgery (94.9; 95.6–99.4) versus conservative treatment (99.3; 98.4–100.2) (*p* = 0.0938). Only 1 patient had recognized diplopia during follow-up.

**Table 2:** HAR% among patients who underwent surgery versus conservative treatment.

Group	Surgery	Conservative treatment	*p*
	Mean (95% CI)	Mean (95% CI)	
All patients	96.0 (93.2–98.8)	99.4 (98.8–100.0)	0.0231^a^
Patients aged 0–12 years	97.6 (84.8–100.4)	99.7 (99.5–99.9)	0.125
Patients aged 13–18 years	94.9 (95.6–99.4)	99.3 (98.4–100.2)	0.0938

HAR%, HESS area ratio; 95% CI 95% percent confidence interval.

a *p* < 0.05, Mann–Whitney *U* test.

### Clinical results in the age 0–12 versus 13–18 years groups (eTable 3 in the supplement)

Patients in the age 0–12 years group (ratio = 29.2; 95% CI 12.6–51.1) had a statistically significantly higher probability of requiring urgent release than patients in the age 13–18 years group (2.7; 0.1–14.2) (Odds Ratio = 14.2; 95% CI 1.62–683.4; *p* = 0.0046). On the other hand, there were no statistically significant differences in follow-up HAR% by age group (98.8; 97.6–100.0 vs. 97.5; 95.6–99.3) (*p* = 0.559).

### Diplopia during follow-up among patients in this study and previous studies (Table 3)

Table 3 includes 10 previous studies about operative pediatric blowout fractures and this study. The duration from injury to the start of urgent repair surgery in this study was shortest (mean ± SD, 11.78 ± 7.74 h; range, 4–24 h). In this study, 96% (95% CI 79.6%–99.9%) of patients who underwent surgery did not have postoperative diplopia at the time of ocular movement fixation. Table 3 shows 5 previous studies that included patients who underwent conservative treatment. The ratio of patients treated with surgery versus conservative treatment in this study was similar to those of previous studies.

**Table 3:** Summary of studies about pediatric blowout fracture. Includes previous case series of pediatric blowout fractures published after 2001 identified in PubMed using the search terms “pediatric, blowout, fracture” on May 31, 2021.

Authors, year	Number of patients who underwent surgery	Number of patients who received conservative treatment	Age	Actual period to urgent repair	Proportion of patients with no postoperative diplopia (%)	Follow-up duration
Bansagi et al., 2000	11	23	≤18 years	13.36 ± 3.45 (0–40) days^b^	NA	NA
Grant et al., 2002	17^a^	2^a^	≤16 years	Mean 8.9 days	82.4	Mean 6.2 months
Lane et al., 2007	30	0	≤20 years	Within 48–72 h	80	>3 months
Tse et al., 2007	4^a^	1^a^	≤19 years	9.75 ± 4.21 (0–19) days^b^	50	1.5–22 months, median 5.5 months
Chi et al., 2010	144	0	≤18 years	NA	97.2	≥6 months
Wang et al., 2010	38	0	≤17 years	Within 48 h	44.7	11 ± 8.7 (4–26) months^b^
Heggie et al., 2015	9	13	≤13 years	Within 24 h	66.7	1–18 months
Su et al., 2015	83	0	≤18 years	NA	83.5	>12 months
Su et al., 2016	135	0	≤18 years	Median 15 days	83.7	>12 months
Koryczan et al., 2021	178	22	≤18 years	NA	NA	1.5–18 months
Current study, 2023	25	36	≤18 years	11.78 ± 7.74 (4–24) h^b^	96	Mean 5 (1–31) months

hr, hour; NA, Not Applicable.

a Only linear fractures.

b Mean ± SD (range).

### Adverse events

No adverse events occurred in this study.

## Discussion

We have shown that we had good outcomes for pediatric orbital blowout fractures based on the algorithm. This simple and effective algorithm will help specialists and non-specialists who refer pediatric orbital blowout fractures to specialists at the appropriate time.

Follow-up ocular movement was satisfactory among patients who underwent surgery, such as urgent release and repair, and patients who received conservative treatment in this study. Ocular movement was evaluated with HAR%, which is an objective measurement.^31^ Although follow-up HAR% was statistically significantly better for patients who received conservative treatment than for patients who underwent surgery, follow-up HAR% for patients who underwent surgery was markedly higher than 85%. If HAR% is more than 85%, diplopia is not recognized.^31^

The proportion of patients without postoperative diplopia in this study was also satisfactory compared with that of previous studies.^3^, ^6^, ^8^, ^11^^,^^12^, ^14^, ^20^, ^27^^,^^28^, ^32^ It would be convenient and reasonable to select treatment according to the algorithm. According to our results, the desirable surgical timing for urgent cases might be within 12 h.

We divided the patients into 2 groups: age 0–12 years and age 13–18 years, similar to a previous report,^6^ in consideration of the growth of the paranasal sinuses.^38^ Regarding the clinical results of the 2 groups, there were statistically significantly more urgent cases in the age 0–12 years group than in the age 13–18 years group. On the other hand, follow-up HAR% was similar in both groups. Therefore, there were no differences in clinical results by age group after treatment according to the algorithm.

The first important feature of the algorithm that differs from previous reports^3^, ^6^ is that the HESS screen test was not used to determine urgency. Pediatric patients, especially patients under 5 years of age, are often uncooperative with the examination.^6^ Therefore, we focused on objective signs to evaluate urgency, such as missing rectus^9^, ^33^ and OCR causing nausea, vomiting, or adynamia.^1^, ^34^, ^35^, ^36^ However, it was essential to confirm the presence of diplopia in order to judge a case as urgent because the symptoms of OCR are similar to those of concussion.

The second important feature of the algorithm is the distribution of surgical timing; urgent release was performed as urgent surgery and repair was performed as standby surgery. The urgent release group included patients who had serious injury of the orbital contents. The repair group included patients who had restricted ocular movement or enophthalmos, but surgery was not urgent. We decided to perform urgent release as soon as possible. Because entrapped orbital contents are severely affected by insufficient blood supply, fibrosis and muscle dysfunction could appear in 6–8 h after entrapment.^7^, ^9^, ^10^, ^33^ Indications for standby surgery are the same as previous studies, such as > 2 mm enophthalmos, extraocular muscle restriction with symptomatic diplopia (within 30° fixation), and large orbital wall defect (>50%) on CT after improvement of orbital swelling.^2^, ^8^, ^11^, ^19^^,^^22^, ^28^, ^34^ Although previous studies have recommended standby surgery within 2 weeks to avoid adhesions among orbital contents,^3^, ^7^, ^8^ standby surgery was performed after ocular movement improvement became stable in the current study. As a result, the timing of repair was within 2 weeks in this study.

The transorbital approach through a subciliary incision or transconjunctival incision is a suitable surgical approach for pediatric orbital blowout floor fractures because it is more convenient to release the incarcerated orbital contents with a transorbital approach in pediatric patients, in whom linear fractures are common.^2^, ^6^ Reconstruction of the orbital floor is rarely needed, especially for linear fractures.^39^ In this study, all patients in the urgent release group except for 1 patient with medial rectus muscle entrapment underwent surgery via the transorbital approach without reconstruction.

Even in patients with limited ocular movement, attention should be paid to conditions such as extraocular muscle palsy, ruptured globe, or retinal detachment that require postponement or avoidance of surgical treatment.^1^, ^40^ All the participants of this study were evaluated by an ophthalmologist to rule out those conditions.

CT is the most suitable modality for pediatric blowout fractures, especially for urgent conditions. The time for imaging is short and it can be performed in pediatric patients.^34^ In addition to the condition of the bone, the condition of the orbital contents can be evaluated in the soft tissue window. On the other hand, Magnetic Resonance Imaging (MRI) has several advantages compared with CT: no radiation, better evaluation of soft tissue, and functional evaluation of orbital contents.^1^, ^41^ In the algorithm, CT, which has the characteristic of short imaging time, was adopted as the first imaging modality. In this study, all patients were able to undergo urgent CT evaluation of orbital conditions. MRI was performed subsequently as needed in non-urgent cases.

We have typically used a subciliary incision for the transorbital approach. However, a transconjunctival incision also could be used; transconjunctival incisions have been reported to result in fewer complications than subciliary incisions.^42^ In this study, the transnasal endoscopic approach was used for non-linear fractures. This approach is relatively common, and postoperative ocular movement are unlikely to differ substantially depending on the type of approach.

Our study has three limitations. First, this was a single-arm study in which delayed surgery in urgent cases was not evaluated. However, more importantly, the treatment of pediatric blowout fractures according to the current algorithm resulted in satisfactory outcomes. Second, this study was conducted at a single institution. Therefore, the number of cases was small. It is necessary to examine more cases in more institutions. Third, the superiority of the treatment was not statistically evaluated because it was not statistically compared to treatments in previous studies. In addition, it is possible that diplopia in special eye positions could not be evaluated due to diplopia.

Despite these limitations, we believe that this study is valuable because it clearly defines the symptom of patients with pediatric orbital blowout fractures who should be selected for urgent release and describes how soon surgery should be performed after trauma. The timing of surgery has been controversial in previous reports: as soon as possible,^9^, ^10^, ^16^, ^20^ within 24 h,^1^, ^7^, ^13^ within 48 h,^2^, ^4^, ^28^ or over 48 h^11^, ^12^, ^17^, ^18^^,^^23^, ^29^ from injury.

## Conclusion

This study has shown the validity of our algorithm. This simple algorithm might be helpful to physicians treating pediatric orbital blowout fractures.

## Conflicts of interest

The authors declare no conflicts of interest.

## References

[1] Chung S.Y., Langer P.D. (2017). Pediatric orbital blowout fractures. Curr Opin Ophthalmol.

[2] Joshi S., Kassira W., Thaller S.R. (2011). Overview of pediatric orbital fractures. J Craniofac Surg.

[3] Su Y., Shen Q., Lin M., Fan X. (2016). Predictive factors for residual diplopia after surgical repair in pediatric patients with orbital blowout fracture. J Craniomaxillofac Surg.

[4] Su Y., Shen Q., Bi X., Lin M., Fan X. (2019). Delayed surgical treatment of orbital trapdoor fracture in paediatric patients. Br J Ophthalmol.

[5] Broyles J.M., Jones D., Bellamy J., Elgendy T., Sebai M., Susaria S.M. (2015). Pediatric orbital floor fractures: outcome analysis of 72 children with orbital floor fractures. Plast Reconstr Surg.

[6] Su Y., Shen Q., Lin M., Fan X. (2015). Diplopia of pediatric orbital blowout fractures: a retrospective study of 83 patients classified by age groups. Medicine (Baltimore).

[7] Gerber B., Kiwanuka P., Dhariwal D. (2013). Orbital fractures in children: a review of outcomes. Br J Oral Maxillofac Surg.

[8] Heggie A.A., Vujcich N.J., Shand J.M., Bordbar P. (2015). Isolated orbital floor fractures in the paediatric patient: case series and review of management. Int J Oral Maxillofac Surg.

[9] Yano H., Suzuki Y., Yoshimoto H., Mimasu R., Hirano A. (2010). Linear-type orbital floor fracture with or without muscle involvement. J Craniofac Surg.

[10] Sugamata A., Yoshizawa N., Shimanaka K. (2013). Timing of operation for blowout fractures with extraocular muscle entrapment. J Plast Surg Hand Surg.

[11] Wang N.C., Ma L., Wu S.Y., Yang F.R., Tsai Y.J. (2010). Orbital blow-out fractures in children: characterization and surgical outcome. Chang Gung Med J.

[12] Lane K., Penne R.B., Bilyk J.R. (2007). Evaluation and management of pediatric orbital fractures in a primary care setting. Orbit.

[13] Gerbino G., Roccia F., Bianchi F.A., Zavattero E. (2010). Surgical management of orbital trapdoor fracture in a pediatric population. J Oral Maxillofac Surg.

[14] Chi M.J., Ku M., Shin K.H., Baek S. (2010). An analysis of 733 surgically treated blowout fractures. Ophthalmologica.

[15] Leitch R.J., Burke J.P., Strachan I.M. (1990). Orbital blowout fractures—the influence of age on surgical outcome. Acta Ophthalmol (Copenh).

[16] de Man K., Wijngaarde R., Hes J., de Jong P.T. (1991). Influence of age on the management of blow-out fractures of the orbital floor. Int J Oral Maxillofac Surg.

[17] Jordan D.R., Allen L.H., White J., Harvey J., Pashby R., Esmaeli B. (1998). Intervention within days for some orbital floor fractures: the white-eyed blowout. Ophthalmic Plast Reconstr Surg.

[18] Egbert J.E., May K., Kersten R.C., Kulwin D.R. (2000). Pediatric orbital floor fracture: direct extraocular muscle involvement. Ophthalmology.

[19] Hatton M.P., Watkins L.M., Rubin P.A. (2001). Orbital fractures in children. Ophthalmic Plast Reconstr Surg.

[20] Grant J.H., Patrinely J.R., Weiss A.H., Kierney P.C., Gruss J.S. (2002). Trapdoor fracture of the orbit in a pediatric population. Plast Reconstr Surg.

[21] Cohen S.M., Garrett C.G. (2003). Pediatric orbital floor fractures: nausea/vomiting as signs of entrapment. Otolaryngol Head Neck Surg.

[22] Baek S.H., Lee E.Y. (2003). Clinical analysis of internal orbital fractures in children. Korean J Ophthalmol.

[23] Yoon K.C., Seo M.S., Park Y.G. (2003). Orbital trapdoor fracture in children. J Korean Med Sci.

[24] Theologie-Lygidakis N., Iatrou I., Alexandridis C. (2007). Blow-out fractures in children: six years’ experience. Oral Surg Oral Med Oral Pathol Oral Radiol Endod.

[25] Parbhu K.C., Galler K.E., Li C., Mawn L.A. (2008). Underestimation of soft tissue entrapment by computed tomography in orbital floor fractures in the pediatric population. Ophthalmology.

[26] Carroll S.C., Ng S.G. (2010). Outcomes of orbital blowout fracture surgery in children and adolescents. Br J Ophthalmol.

[27] Tse R., Allen L., Matic D. (2007). The white-eyed medial blowout fracture. Plast Reconstr Surg.

[28] Bansagi Z.C., Meyer D.R. (2000). Internal orbital fractures in the pediatric age group: characterization and management. Ophthalmology.

[29] Kim J., Lee H., Chi M., Park M., Lee J., Baek S. (2010). Endoscope-assisted repair of pediatric trapdoor fractures of the orbital floor: characterization and management. J Craniofac Surg.

[30] Cope M.R., Moos K.F., Speculand B. (1999). Does diplopia persist after blow-out fractures of the orbital floor in children?. Br J Oral Maxillofac Surg.

[31] Furuta M., Yago K., Iida T. (2006). Correlation between ocular motility and evaluation of computed tomography in orbital blowout fracture. Am J Ophthalmol.

[32] Koryczan P., Zapała J., Gontarz M., Wyszyńska-Pawelec G. (2021). Surgical treatment of enophthalmos in children and adolescents with pure orbital blowout fracture. J Oral Sci.

[33] Yano H., Minagawa T., Masuda K., Hirano A. (2009). Urgent rescue of’ missing rectus’ in blowout fracture. J Plast Reconstr Aesthet Surg.

[34] Wei L.A., Durairaj V.D. (2011). Pediatric orbital floor fractures. J Aapos.

[35] Dunphy L., Anand P. (2019). Paediatric orbital trapdoor fracture misdiagnosed as a head injury: a cautionary tale!. BMJ Case Rep.

[36] Kosaka M., Sakamoto T., Yamamichi K., Yamashiro Y. (2014). Different onset pattern of oculocardiac reflex in pediatric medial wall blowout fractures. J Craniofac Surg.

[37] Kanda Y. (2013). Investigation of the freely available easy-to-use software’ EZR’ for medical statistics. Bone Marrow Transplant.

[38] Jones L.C., Flint R.L., Kushner G.M., Jones L.C. (2021). Pediatric maxillofacial trauma.

[39] Sugamata A., Yoshizawa N. (2015). A case of blowout fracture of the orbital floor in early childhood. Int Med Case Rep J.

[40] Young S.M., Koh Y.T., Chan E.W., Amrith S. (2018). Incidence and risk factors of inferior rectus muscle palsy in pediatric orbital blowout fractures. Craniomaxillofac Trauma Reconstr.

[41] Morotomi T., Iuchi T., Hashimoto T., Sueyoshi Y., Nagasao T., Isogai N. (2015). Image analysis of the inferior rectus muscle in orbital floor fracture using cine mode magnetic resonance imaging. J Craniomaxillofac Surg.

[42] Al-Moraissi E.A., Thaller S.R., Ellis E. (2017). Subciliary vs. transconjunctival approach for the management of orbital floor and periorbital fractures: a systematic review and meta-analysis. J Craniomaxillofac Surg.

