# Supplementary material for: Algorithm for pediatric orbital blowout fractures: a 20-year retrospective cohort study
Source: Braz J Otorhinolaryngol. 2023 Jan 27;89(3):447–55. doi: 10.1016/j.bjorl.2023.01.004 (PMC10164772; doi:10.1016/j.bjorl.2023.01.004)
Supplement: Supplementary file 1 [file mmc1.docx]

BJORL-D-22-00300 - **Supplement data**

| **Patient categorization according to the algorithm** |
| --- |
| eFigure 1 in the supplement shows the results of patient categorization based on the algorithm. |
| *All patients* (eFigure 1A in the supplement) |
| Of 61 patients, 33 had linear fractures and 28 had non-linear fractures. Of the 33 patients with linear fractures, 9 had both diplopia and an urgent sign, such as missing rectus or OCR, and underwent urgent release surgery as soon as possible within 24 hours from the time of the injury. The other 24 patients with linear fractures and the 28 patients with non-linear fractures were categorized as standby. Their ocular movement was evaluated after fixation of eye movement and decreased orbital swelling. Of the 52 standby patients, 15 underwent repair surgery due to recognition of diplopia and restriction of ocular movement on the HESS screen test. One of the other 37 standby patients underwent repair surgery due to recognition of enophthalmos without diplopia. The remaining 36 standby patients received conservative treatment without surgical repair. |
| *Patients in the age 0–12 years group* (eFigure 1B in the supplement) |
| According to the algorithm, of the 24 patients aged 0–12 years, 7 were categorized as urgent release, 3 as repair, and 14 as conservative treatment. |
| *Patients in the age 13–18 years group* (eFigure 1C in the supplement) |
| According to the algorithm, of the 37 patients aged 13–18 years, 1 was categorized as urgent release, 14 as repair, and 22 as conservative treatment. |
| **The characteristics of the age 0**–**12 years group** |
| *Overall* |
| Of the 24 patients, 18 were male and 6 were female. Median age was 10 years, with range of 5–12 years. Mean follow-up duration was 108.5 days (range, 1–605 days). None had symptoms such as decreased initial visual acuity or enophthalmos on presentation, or enophthalmos on follow-up. Mean HAR% on follow-up was 98.8 (98.8–100.0) (mean [95% CI]). |
| *Patients who underwent surgery* |
| In the age 0–12 years group, 10 patients underwent reconstructive surgery. There were 6 patients with missing rectus and 6 patients with OCR, of whom 4 patients had both missing rectus and OCR. Two patients had only missing rectus and 2 patients had only OCR. None had postoperative diplopia. |
| *Patients who received conservative treatment* |
| In the age 10–12 years group, 14 patients were followed without surgery. None had missing rectus; only 1 patient had OCR. None had diplopia during follow-up. |
| **The characteristics of the age 13**–**18 years group** |
| *Overall* |
| Of 37 patients, 34 were male and 3 were female. Median age was 16 (range, 13–18) years. Mean follow-up duration was 132.1 days (range, 1–922 days). None of the patients had symptoms such as decreased visual acuity initially or enophthalmos on follow-up, except for 1 patient who presented with enophthalmos. Mean HAR% on follow-up was 97.5 (95.6‒99.4) (mean [95% CI]). |
| Patients who underwent surgery |
| In the age 13–18 years group, 15 patients underwent surgery. There was 1 patient with missing rectus and 2 patients with OCR, of whom 1 patient had both missing rectus and OCR. One patient had only OCR. None had postoperative diplopia. |
| **Patients who received conservative treatment** |
| In the age 13–18 years group, 22 patients were followed without surgery. None had missing rectus or OCR. None had diplopia during follow-up. |

**eTable 1** Characteristics of patients in the age 0–12 years group.

| **Characteristic** | **Total patient** | **Operative patient** | **Conservative patient** |
| --- | --- | --- | --- |
|  | **Value (%)** | **Value (%)** | **Value (%)** |
| Sex |  |  |  |
| Male | 18 (75) | 7 (0) | 11 (79) |
| Female | 6 (25) | 3 (30) | 3 (21) |
| Age, yr |  |  |  |
| Median | 10 | 9 | 10 |
| Range | 5‒12 | 7‒12 | 5‒12 |
| Follow-up, day |  |  |  |
| Average | 108.5 | 187.3 | 52.2 |
| Range | 1‒605 | 27‒605 | 1‒587 |
| Decreased initial visual acuity | 0/24 (0) |  |  |
| Enophthalmos on presentation | 0/24(0) |  |  |
| Enophthalmos on follow-up | 0/24 (0) |  |  |
| Surgical management | 10/24 (42) |  |  |
| Missing rectus |  | 6/10 (60) | 0/14 (0) |
| OCR |  | 6/10 (60) | 1/14(7) |
| Urgent release |  | 7/10 (70) |  |
| Diplopia on follow-up |  | 0/10 (0) | 0/14(0) |
| HARS% on follow-up |  |  |  |
| Mean | 98.8 | 97.6 | 99.7 |
| 95% CI | 97.6‒100.0 | 84.8‒100.4 | 99.5‒99.9 |

OCR, Oculocardiac Reflex; HAR%, Hess Area Ratio; 95% CI, 95% Percent Confidence Interval.

**eTable 2** Characteristics of patients in the age 13–18 years group.

| **Characteristic** | **Total patient** | **Operative patient** | **Conservative patient** |
| --- | --- | --- | --- |
|  | **Value (%)** | **Value (%)** | **Value (%)** |
| Sex |  |  |  |
| Male | 34 (92) | 13 (87) | 21 (95) |
| Female | 3 (8) | 2 (13) | 1 (5) |
| Age, yr |  |  |  |
| Median | 16 | 16 | 16 |
| Range | 13‒18 | 13‒1 | 13‒18 |
| Follow-up, day |  |  |  |
| Average | 132.1 | 279.9 | 31.3 |
| Range | 1‒922 | 43‒922 | 1‒407 |
| Decreased initial visual acuity | 0/37 (0) |  |  |
| Enophthalmos on presentation | 1/37 (3) |  |  |
| Enophthalmos on follow-up | 0/37 (0) |  |  |
| Surgical management | 15/37 (41) |  |  |
| Missing rectus |  | 1/15 (7) | 0/22 (0) |
| OCR |  | 2/15 (13) | 0/22 (0) |
| Urgent release |  | 1/15 (7) |  |
| Diplopia on follow-up |  | 1/15 (7) | 0/22 (0) |
| HARS% on follow-up |  |  |  |
| Mean | 97.5 | 94,9 | 99.3 |
| 95% CI | 95.6‒99.4 | 90.1‒99.3 | 98.4‒100.2 |

OCR, Oculocardiac Reflex; HAR%, Hess Area Ratio; 95% CI, 95% Percent Confidence Interval.

**eTable 3** Clinical results in the age 0–12 years versus age 13–18 years groups.

| **Variable** | **Age 0‒12 years group** | **Age 13‒18 years group** | **p** |
| --- | --- | --- | --- |
|  | **Value** | **Value** |  |
| Urgent release (ratio [95% CI])] | 7 (29.2 [12.6‒51.1]) | 1 (2.7 [0.1‒14.2]) | 0.0046^a^ |
| HARS% (95% CI) | 98.8 (97.6‒1000) | 97.5 (95.6‒99.3) | 0.559 |

Significance of data (^a^ *p* < 015; significance indicated by p-value derive from Fisher's exact test and Mann-Whitney *U* tes).

95% CI, 95% Percent Confidence Interval; HAR%, HESS Area Ratio.

**eFigure 1** Patient categorization according to the algorithm. (A) Distribution of patients according to the algorithm into the urgent release (n = 6), repair (n = 16), and conservative treatment (n = 36) categories. (B) Distribution of patients in the age 0–12 years group according to the algorithm into the urgent release (n = 7), repair (n = 3), and conservative treatment (n = 14) categories. (C) Distribution of patients in the age 13–18 years group according to the algorithm into the urgent release (n = 1), repair (n = 14), and conservative treatment (n = 22) categories. OCR, Oculocardiac Reflex.

**
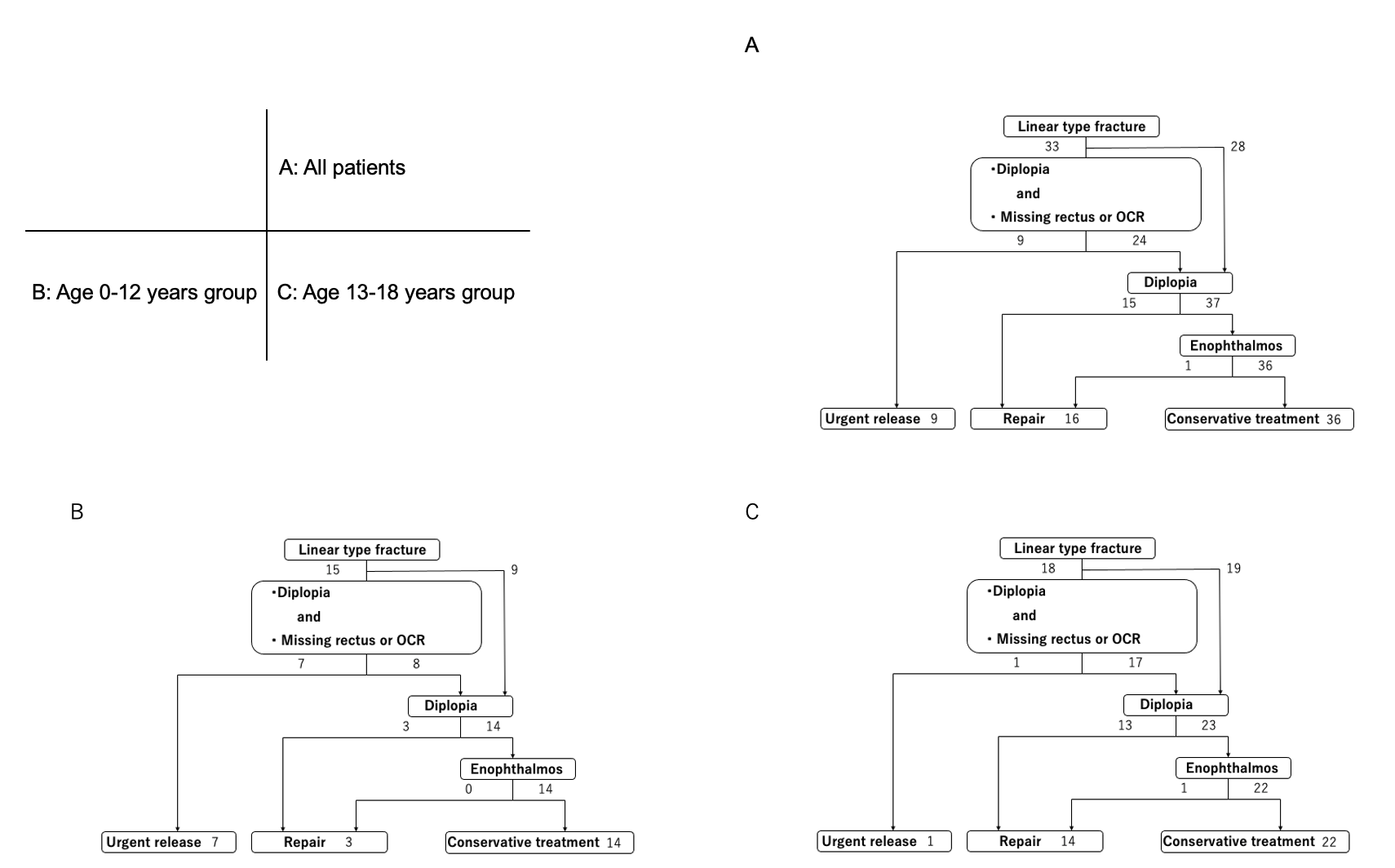
**
